# Water Deficit and Rehydration Reveal Genotypic Differences in Apple Tree Physiological Performance

**DOI:** 10.3390/plants15081179

**Published:** 2026-04-10

**Authors:** Frantisek Hnilicka, Tomáš Rýgl, Pavol Suran, Lubor Zelený, Naz Akgűn

**Affiliations:** 1 Czech University of Life Sciences Prague, Faculty of Agrobiology, Food and Natural Resources, Department of Botany and Plant Physiology, Kamýcká 129, Suchdol, 165 21 Prague, Czech Republic; 2 Research and Breeding Institute Pomology Holovousy Ltd., Holovousy 129, 50801 Hořice v Podkrkonoší, Czech Republic; 3 Ondokuz Mayıs University, Department of Field Crops, 55100 Samsun, Türkiye

**Keywords:** water deficit, apple tree, *Malus domestica*, gas exchange, pigments, chlorophyll fluorescence

## Abstract

The apple tree (*Malus domestica* Borkh.) is one of the most economically important fruit crops worldwide, and its productivity is increasingly affected by water deficit. Understanding genotype-specific physiological responses to water deficit is important for improving water deficit resilience in apple. This study evaluated the effects of water deficit (14 days) and subsequent rehydration (7 days) on eight apple genotypes (‘Galaval’, ‘Idared’, ‘Rubinstep’, ‘B11’, ‘HL 308’, ‘HL 2010’, ‘HL 2350’, and ‘HL 827’) grown under semi-controlled container conditions. Physiological parameters, including pigment content, gas exchange, chlorophyll fluorescence, water use efficiency, and leaf water status, were assessed. Water deficit affected all measured parameters, with responses differing among genotypes. In most cases, water deficit was associated with reduced gas exchange and chlorophyll fluorescence, as well as changes in pigment content and leaf water status. However, the magnitude and direction of these responses varied depending on genotype. Some genotypes (e.g., ‘HL 2350’ and ‘B11’) showed more stable physiological performance under water deficit conditions, while others (e.g., ‘Idared’ and ‘Rubinstep’) exhibited more pronounced changes. Rehydration resulted in partial recovery of physiological parameters, although values generally did not reach control levels within the experimental period. The results indicate substantial genotypic variability in physiological responses to short-term water deficit under controlled conditions. These findings provide useful information for further research on water deficit responses in apple; however, additional studies under field conditions and including growth and yield parameters are required to assess the agronomic relevance of the observed differences.

## 1. Introduction

Drought is one of the most important environmental factors limiting plant productivity worldwide and is expected to increase in frequency and intensity under ongoing climate change, posing a significant challenge for sustainable fruit production systems [1,2]. Plant responses to water deficit vary depending on species, developmental stage, and stress duration, and involve complex physiological and biochemical adjustments [3,4,5].

Apple (*Malus domestica* Borkh.) is among the most economically important fruit crops worldwide. According to FAOSTAT (2025) [6], the global area of apple cultivation reached 4,685,316 ha in 2024, with the largest share in Asia (3,311,614 ha) and the smallest in Oceania (28,032 ha). Apple yields vary considerably among regions, ranging from approximately 19.16 t ha^−1^ in Europe to 31.78 t ha^−1^ in the Americas, with global production approaching 98 million tons. However, apple is considered relatively sensitive to water deficit, which negatively affects growth, yield, and fruit quality [7,8].

Apple production is particularly sensitive to water availability due to high transpiration demands and relatively shallow root systems, especially when grafted onto dwarfing rootstocks such as M9 [9,10]. Water deficit in apple trees is commonly associated with reduced stomatal conductance, decreased photosynthetic activity, impaired chlorophyll fluorescence, and changes in pigment composition [11,12,13,14,15]. These responses are often accompanied by altered plant water status and increased production of reactive oxygen species, which can further disrupt cellular processes and photosynthetic efficiency [16,17]. These processes are closely linked to changes in photosynthetic performance and chlorophyll fluorescence, which are widely used as indicators of stress-induced damage to the photosynthetic apparatus [18,19].

Previous studies have reported considerable variability among apple genotypes in their responses to water deficit, reflecting differences in physiological regulation and adaptive capacity [10,14,15]. Genotypes with improved water deficit tolerance often maintain higher photosynthetic activity, more stable chlorophyll fluorescence, and more efficient water use under stress conditions [20,21]. Such variability represents an important resource for breeding and selection of drought-resilient plant material.

The plant material used in this study includes both commercial cultivars (‘Galaval’, ‘Idared’, ‘Rubinstep’) and advanced breeding lines (‘B11’, ‘HL 308’, ‘HL 2010’, ‘HL 2350’, and ‘HL 827’), allowing comparison of established cultivars with newly developed genotypes during water deficit conditions.

In addition to drought responses, the ability of plants to recover following rehydration represents a critical but less explored aspect of stress physiology, particularly in woody fruit species [22,23].

Despite extensive research on water deficit responses in apple, studies integrating multiple physiological parameters across genotypes during both water deficit and subsequent rehydration remain limited, particularly under controlled experimental conditions [21].

Therefore, the aim of this study was to evaluate genotype-dependent differences in physiological responses of apple trees to water deficit and subsequent rehydration under semi-controlled conditions. It was hypothesized that (i) apple genotypes differ in their physiological responses to water deficit, and (ii) water deficit significantly alters gas exchange, pigment content, chlorophyll fluorescence, and plant water status, with genotype-specific recovery following rehydration.

## 2. Results

### 2.1. Content of Pigments

**Figure 1:**
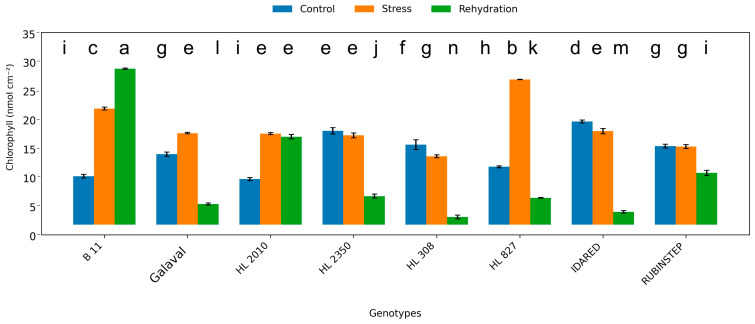
Total chlorophyll content (nmol cm^−2^) in leaves of apple genotypes under control conditions, water deficit, and subsequent rehydration. Bars represent mean values ± standard deviation (SD) (*n* = 4). Different letters indicate statistically significant differences among treatments within each genotype (Tukey’s HSD test, *p* ≤ 0.05).

Total chlorophyll content exhibited genotype-specific responses to water deficit (Figure 1). Under control conditions, chlorophyll concentrations ranged between approximately 9 and 18 nmol cm^−2^. Water deficit altered chlorophyll content in a genotype-dependent manner. While reductions were observed in several genotypes, others maintained comparable values or showed slight increases in response to water deficit conditions. However, selected genotypes, particularly ‘HL 2350’ and ‘B11’, maintained relatively stable chlorophyll levels, suggesting a relatively more stable chlorophyll response under the imposed conditions.

**Figure 2:**
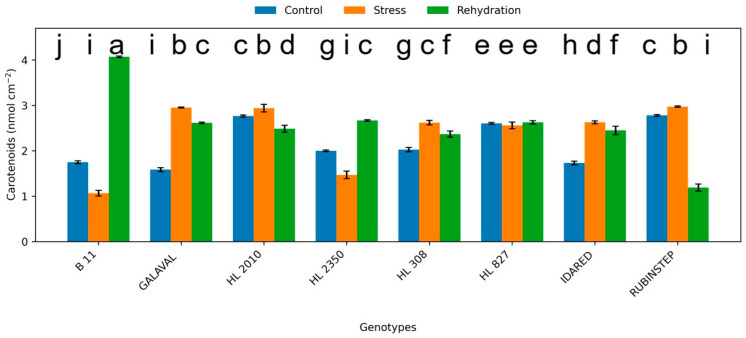
Carotenoid content (nmol cm^−2^) in leaves of apple genotypes under control conditions, water deficit, and subsequent rehydration. Bars represent mean values ± standard deviation (SD) (*n* = 4). Different letters indicate statistically significant differences among treatments within each genotype (Tukey’s HSD test, *p* ≤ 0.05).

Carotenoid content also showed variable responses among genotypes (Figure 2), with decreases observed in some cases and stable values in others. While water deficit-induced reductions were observed in some genotypes, others maintained stable carotenoid levels, indicating potential protective roles against oxidative stress. The relatively stable pigment composition observed in ‘HL 2350’ and ‘HL 827’ suggests more efficient photoprotective mechanisms compared with more sensitive genotypes such as ‘Idared’.

### 2.2. Leaf Gas Exchange 

**Figure 3:**
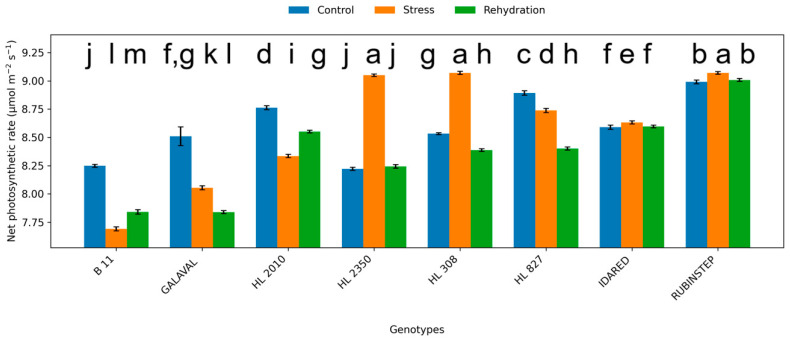
Net photosynthetic rate (Pn; µmol CO_2_ m^−2^ s^−1^) in leaves of apple genotypes under control conditions, water deficit, and subsequent rehydration. Bars represent mean values ± standard deviation (SD) (*n* = 4). Different letters indicate statistically significant differences among treatments within each genotype (Tukey’s HSD test, *p* ≤ 0.05).

**Figure 4:**
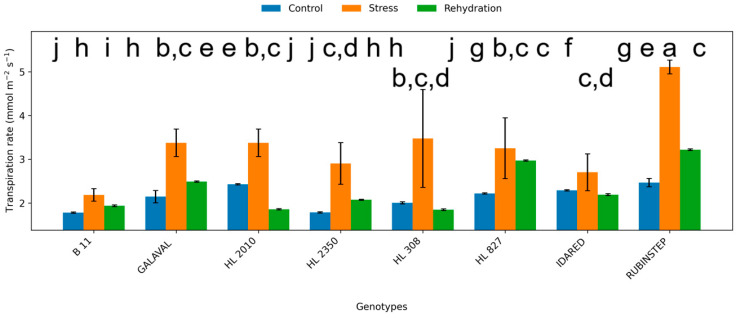
Transpiration rate (E; mmol H_2_O m^−2^ s^−1^) in leaves of apple genotypes under control conditions, water deficit, and subsequent rehydration. Bars represent mean values ± standard deviation (SD) (*n* = 4). Different letters indicate statistically significant differences among treatments within each genotype (Tukey’s HSD test, *p* ≤ 0.05).

**Figure 5:**
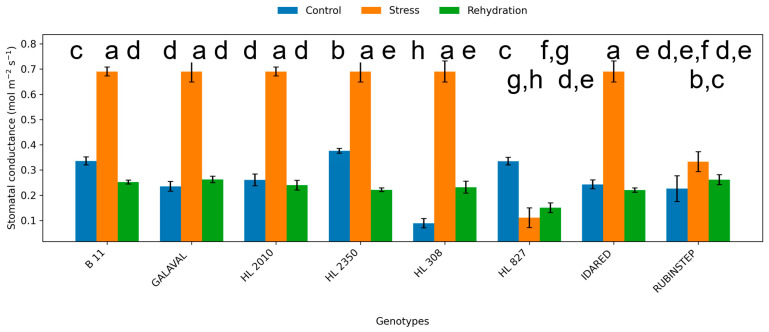
Stomatal conductance (gs; mol H_2_O m^−2^ s^−1^) in leaves of apple genotypes under control conditions, water deficit, and subsequent rehydration. Bars represent mean values ± standard deviation (SD) (*n* = 4). Different letters indicate statistically significant differences among treatments within each genotype (Tukey’s HSD test, *p* ≤ 0.05).

Water deficit significantly affected gas exchange parameters, including net photosynthetic rate (Pn), transpiration rate (E), and stomatal conductance (gs) (Figure 3, Figure 4 and Figure 5). Under control conditions, Pn ranged from approximately 7.5 to 9.0 µmol CO_2_ m^−2^ s^−1^. In several genotypes, water deficit was associated with a reduction in Pn; however, in other cases values remained comparable to control levels or showed only minor variation.

The magnitude of reduction was genotype-dependent. ‘HL 2350’, ‘B11’, and partially ‘HL 308’ maintained higher photosynthetic activity under water deficit conditions, suggesting relatively smaller changes in photosynthetic performance. In contrast, ‘Idared’ and ‘Rubinstep’ exhibited a pronounced decline in Pn, suggesting higher sensitivity to water deficit.

Transpiration rate and stomatal conductance showed variable responses, and their patterns were not consistent across all genotypes. Changes in stomatal conductance under water deficit conditions varied among genotypes and did not show a consistent pattern. However, genotypes such as ‘HL 2350’ maintained moderate gs values, allowing continued carbon assimilation while limiting excessive water loss.

Following rehydration, partial recovery of Pn, E, and gs was observed across all genotypes. However, values generally remained comparable to or slightly below control levels, depending on genotype, and did not fully return to control levels within the 7-day period, indicating persistent effects of water deficit on photosynthetic performance.

### 2.3. Water Use Efficiency

**Figure 6:**
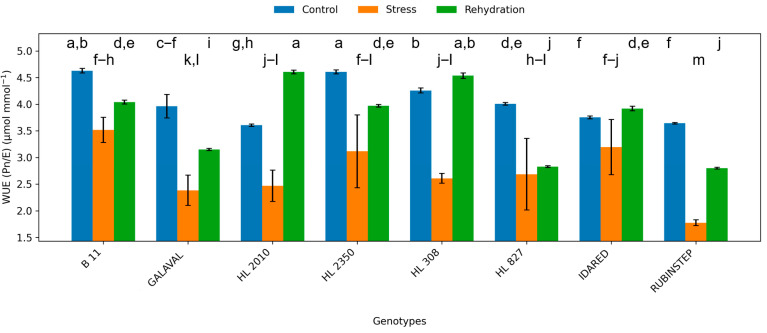
Water use efficiency (WUE; Pn/E) in leaves of apple genotypes under control conditions, water deficit, and subsequent rehydration. Bars represent mean values ± standard deviation (SD) (*n* = 4). Different letters indicate statistically significant differences among treatments within each genotype (Tukey’s HSD test, *p* ≤ 0.05).

Water use efficiency (WUE) responded dynamically to water deficit (Figure 6). In several genotypes, including ‘HL 2350’ and ‘B11’, WUE increased during water deficit conditions, reflecting a more efficient balance between carbon assimilation and water loss. This increase suggests that these genotypes may maintain photosynthetic activity while reducing transpiration.

In contrast, genotypes such as ‘Idared’ showed only minor changes or inconsistent responses in WUE, indicating less effective physiological adjustment to water deficit conditions. Following rehydration, WUE values tended to return toward control levels, although genotype-specific differences remained evident.

### 2.4. Chlorophyll Fluorescence

**Figure 7:**
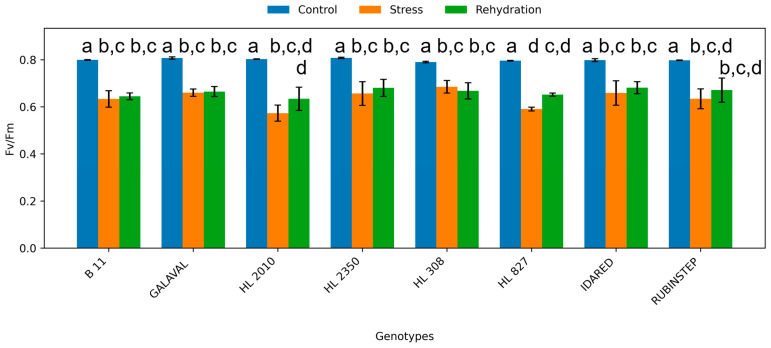
Maximum quantum yield of photosystem II (Fv/Fm) in leaves of apple genotypes under control conditions, water deficit, and subsequent rehydration. Bars represent mean values ± standard deviation (SD) (*n* = 4). Different letters indicate statistically significant differences among treatments within each genotype (Tukey’s HSD test, *p* ≤ 0.05).

**Figure 8:**
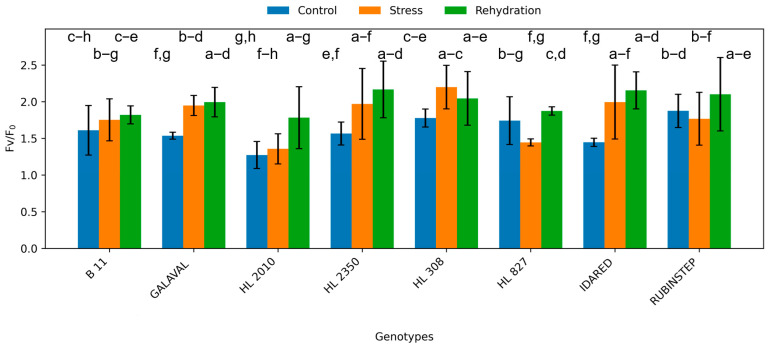
Potential photochemical efficiency of photosystem II (Fv/F_0_) in leaves of apple genotypes under control conditions, water deficit, and subsequent rehydration. Bars represent mean values ± standard deviation (SD) (*n* = 4). Different letters indicate statistically significant differences among treatments within each genotype (Tukey’s HSD test, *p* ≤ 0.05).

Chlorophyll fluorescence parameters were consistently affected by water deficit (Figure 7 and Figure 8). The maximum quantum yield of PSII (Fv/Fm) decreased under water deficit conditions in most genotypes, indicating stress-induced limitations in photochemical efficiency.

The extent of reduction varied among genotypes. Genotypes such as ‘HL 2350’ and ‘HL 827’ showed smaller decreases in Fv/Fm, suggesting better protection of the photosynthetic apparatus. In contrast, more sensitive genotypes showed a pronounced decline, indicating higher susceptibility to photoinhibition.

Rehydration resulted in partial recovery of fluorescence parameters; however, values remained below control levels, confirming that water deficit-induced damage or downregulation of PSII was not fully reversible within the experimental timeframe.

### 2.5. Leaf Water Regime 

**Figure 9:**
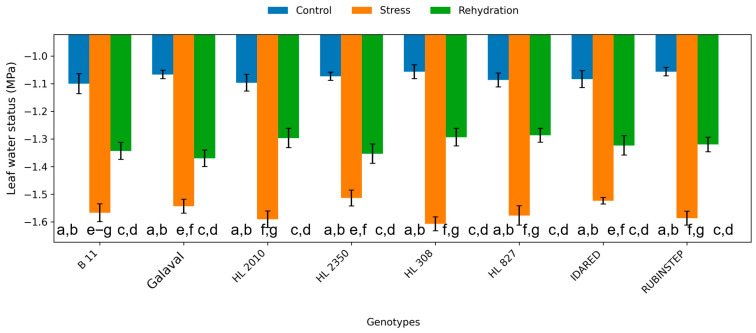
Leaf water status (ψleaf; MPa) in apple genotypes under control, water deficit, and subsequent rehydration. Bars represent mean values ± standard deviation (SD) (*n* = 4). Different letters indicate statistically significant differences among treatments within each genotype (Tukey’s HSD test, *p* ≤ 0.05). Values should be interpreted as relative indicators of leaf water status.

Leaf water status declined during water deficit conditions in all genotypes (Figure 9), confirming the effectiveness of the imposed water deficit. Values decreased from approximately −1.1 MPa under control conditions to around −1.5 MPa in response to water deficit conditions, depending on genotype.

Although all genotypes experienced reduced water status, some genotypes maintained less negative values, indicating a more efficient water management strategy. Following rehydration, partial recovery was observed; however, full restoration to control levels was not achieved.

### 2.6. Principal Component Analysis

**Figure 10:**
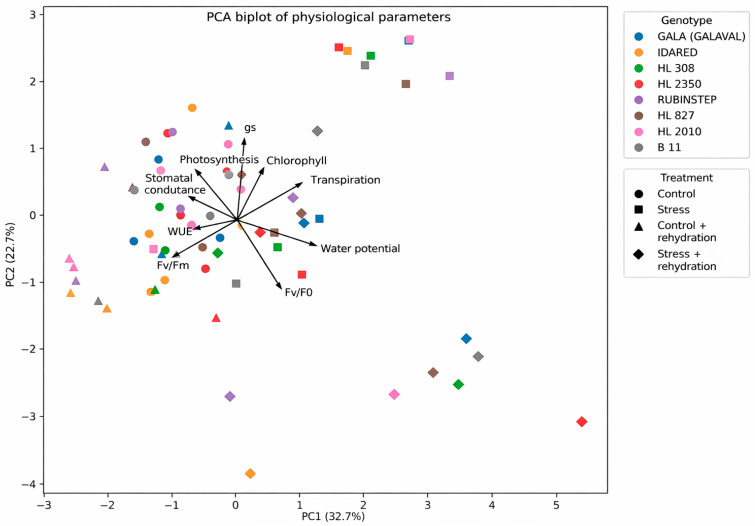
Principal component analysis (PCA) biplot based on physiological parameters. Arrows indicate variable loadings, and points represent individual apple genotypes (‘Galaval’, ‘Idared’, ‘Rubinstep’, ‘B11’, ‘HL 308’, ‘HL 2010’, ‘HL 2350’, and ‘HL 827’) across control, water deficit, and rehydration treatments. The first principal component (PC1) is primarily associated with gas exchange parameters and water use efficiency, while the second component (PC2) reflects variation in chlorophyll fluorescence and pigment-related traits. PC1 and PC2 explain 32.7% and 22.7% of the total variance, respectively.

Principal component analysis (PCA) integrated physiological responses across genotypes and treatments (Figure 10). The first principal component (PC1), explaining 32.7% of the total variance, was primarily associated with gas exchange parameters (Pn, gs, E) and WUE, reflecting a gradient of physiological performance in response to water deficit conditions. The second component (PC2), explaining 22.7% of the variance, was related mainly to chlorophyll fluorescence and pigment content, indicating variation in photochemical efficiency and stress responses.

The PCA biplot revealed that some separation along PC1 can be observed; however, clear clustering of samples according to treatment was not evident. Rehydrated plants occupied intermediate positions, reflecting partial recovery.

Genotype-specific patterns were observed; however, these did not form clearly separated clusters even under water deficit conditions. In contrast, ‘Idared’ and ‘Rubinstep’ were shifted toward regions associated with reduced photosynthetic performance and stress-related responses.

Although complete clustering of genotypes was not observed, the PCA highlights variable physiological responses among genotypes in response to water deficit and rehydration.

## 3. Discussion

This study demonstrated that water deficit significantly affects the physiological performance of apple trees, with responses strongly dependent on genotype. The observed variability among genotypes confirms that water deficit tolerance in apple is a complex trait involving coordinated regulation of gas exchange, photochemical processes, and plant water status.

Water deficit reduced photosynthetic activity, transpiration, and stomatal conductance in most genotypes, indicating limitations in photosynthetic performance; however, the relative contribution of different limiting factors cannot be conclusively determined. Stomatal closure represents an early response to water deficit, restricting CO_2_ uptake and reducing transpiration losses; however, prolonged or more severe stress may also impair biochemical processes associated with carbon assimilation [21,24]. The differential response observed among genotypes suggests variation in the ability to regulate stomatal behavior and maintain photosynthetic function under limited water availability.

Genotypes such as ‘HL 2350’ and ‘B11’ maintained relatively stable photosynthetic rates and stomatal conductance during water deficit conditions, suggesting more stable physiological performance under the imposed conditions. This stability likely reflects a more efficient balance between carbon assimilation and water conservation, as further supported by increased water use efficiency in these genotypes. In contrast, ‘Idared’ and ‘Rubinstep’ exhibited a pronounced decline in gas exchange parameters, suggesting higher sensitivity to water deficit and a reduced capacity to maintain physiological activity under stress.

Changes in chlorophyll fluorescence and pigment content further support these differences in stress response strategies [25]. The decline in Fv/Fm observed under water deficit conditions indicates reduced photochemical efficiency of photosystem II, which is commonly associated with photoinhibition or downregulation of photosynthesis under stress [19,24]. However, smaller reductions in fluorescence parameters in genotypes such as ‘HL 2350’ and ‘HL 827’ suggest more effective protection of the photosynthetic apparatus. Stable carotenoid content in these genotypes may indicate enhanced photoprotective capacity, as carotenoids play a key role in dissipating excess energy and limiting oxidative damage [16,17].

Water use efficiency emerged as an important indicator of water deficit response. The increase in WUE observed in several genotypes in response to water deficit conditions reflects a relatively greater reduction in transpiration compared with photosynthesis, indicating improved intrinsic water-use efficiency. This response has been reported in some studies; however, it should not be interpreted alone as an indicator of overall drought tolerance [21,24]. Not all genotypes exhibited this response, highlighting differences in physiological adjustment mechanisms.

Leaf water status declined in all genotypes under water deficit, confirming the effectiveness of the applied stress [26,27]. Nevertheless, differences in the magnitude of decline suggest variation in water uptake, transport, or retention capacity. Genotypes maintaining less negative water potential values likely possess more efficient hydraulic regulation or osmotic adjustment, contributing to improved water deficit tolerance.

The rehydration phase revealed only partial recovery of physiological parameters, indicating that short-term rewatering was insufficient to fully restore plant function. This incomplete recovery suggests the presence of lasting effects of water deficit, including potential damage to the photosynthetic apparatus or delayed recovery of metabolic processes. Similar patterns have been reported in woody species, where recovery after water deficit is often slower than the onset of stress [23]. The ability to recover following rehydration represents an important but often overlooked component of water deficit tolerance.

The PCA provided an integrated view of physiological responses and confirmed the dominant effect of water deficit on plant performance. The separation of treatments along the first principal component reflects the central role of gas exchange parameters and water use efficiency in determining physiological status in response to water deficit conditions. At the same time, the positioning of genotypes in the PCA space indicates distinct response strategies. Genotypes such as ‘HL 2350’ and ‘B11’ remained closer to control conditions even during water deficit conditions, suggesting relatively smaller shifts in physiological parameters, whereas ‘Idared’ and ‘Rubinstep’ were associated with stress-related responses and reduced physiological performance.

Although complete clustering of genotypes was not observed, the PCA highlights the complexity of genotype-dependent responses and suggests that water deficit tolerance is not governed by a single trait but rather by the integration of multiple physiological processes. This finding is consistent with previous studies emphasizing the multifactorial nature of water deficit tolerance in fruit crops [21].

Several limitations of this study should be acknowledged. The experiment was conducted under semi-controlled container conditions, which may not fully reflect field environments, particularly with respect to root development and soil water dynamics. In addition, the relatively short duration of water deficit and rehydration may not capture long-term adaptive responses. The assessment of leaf water status using expressed sap provides relative rather than absolute values, and therefore results should be interpreted accordingly. Finally, the study focused on physiological parameters and did not include growth or yield measurements, which are essential for evaluating agronomic performance.

Despite these limitations, the results clearly demonstrate genotype-dependent differences in water deficit response and highlight the importance of integrating physiological traits in the evaluation of plant performance under water deficit conditions. The identified genotypes with more stable physiological responses represent promising material for further research and breeding aimed at improving water deficit tolerance in apple.

## 4. Materials and Methods

### 4.1. Experimental Design

The experiment was conducted under partially controlled conditions in a foil-covered facility at the Research and Breeding Institute of Pomology Holovousy Ltd., eastern Bohemia (50.383629° N, 15.576902° E; 360 m a.s.l.). The facility allowed natural light conditions while protecting plants from direct precipitation and enabling controlled irrigation.

The experiment was established in early spring 2023 using one-year-old nursery trees grafted onto M9 rootstock. Trees were grown in 30 L containers filled with a commercial substrate (RKS II; AGRO CS Plc., Říkov, Czech Republic; pH 5.0–6.5; nutrient content: N 80–120 mg L^−1^, P 22–44 mg L^−1^, K 83–124 mg L^−1^), composed of 80% white peat and 20% black peat, supplemented with mineral soil (20 kg m^−3^). The substrate was free of weeds and pests. Containers were arranged at a spacing of 0.5 × 0.6 m.

Two irrigation regimes were applied. Irrigation was provided as a fixed daily dose adjusted to plant size and container conditions. Control plants received a full irrigation dose (15 L per 15 min per day), while the water deficit treatment consisted of 50% of this amount (7.5 L per 15 min per day). The applied irrigation levels were determined in preliminary trials to ensure sufficient water supply in control plants and to induce a measurable water deficit in the stress treatment. This approach is commonly used in container experiments where direct estimation of field evapotranspiration is not applicable.

Substrate moisture was regularly checked during the experiment to maintain consistent differences between treatments, although it was not continuously monitored using sensors. Water deficit was imposed for 14 days (BBCH 55–60), followed by a 7-day rehydration period (BBCH 60–67). Outside the water deficit period, all plants were irrigated at the full rate. Each genotype and treatment included four biological replicates (individual trees). A representative image of the experimental setup is provided in Supplementary Figure S3.

### 4.2. Plant Material

In spring 2021, planting material was tested for viruses and phytoplasmas using ELISA and PCR methods. In summer 2021, scions were grafted onto M9 rootstock and cultivated in a nursery to ensure uniform growth and minimize bud damage. In 2023, one-year-old nursery healthy and morphologically uniform trees were transferred to the foil-covered facility.

The experiment included eight apple genotypes: three commercial cultivars (‘Galaval’, ‘Idared’, ‘Rubinstep’) and five advanced breeding lines (‘B11’, ‘HL 308’, ‘HL 2010’, ‘HL 2350’, and ‘HL 827’).

Four biologically independent trees per genotype and treatment were used. Measurements were conducted at BBCH stages 31, 39, 55 (7th day of stress), 60 (14th day of stress), and 67 (7th day of rehydration).

### 4.3. Physiological Measurements

Measurements were conducted at BBCH stages 31, 39, 55, 60, and 67, corresponding to control, drought, and rehydration phases.

#### 4.3.1. Pigment Content

Chlorophyll a, chlorophyll b, total chlorophyll, and carotenoid contents were determined according to Porra et al. [28]. Leaf discs (1 cm^2^) were sampled from fully expanded apple leaves, placed in plastic vials, and extracted in 1 mL dimethylformamide (DMF; Merck KGaA, Darmstadt, Germany) for 24 h in darkness at low temperature under continuous shaking. Pigment concentrations were measured spectrophotometrically (Evolution 2000 UV–Vis; Thermo Fisher Scientific, Waltham, MA, USA) at wavelengths of 480, 648.8, 663.8, and 710 nm, using DMF as a blank.

The equations for calculating the pigments are as follows:

Chl a = 12.0 A_663.8_ − 3.11 A_646.8_ (1)

Chl b = 20.78 A_646.8_ − 4.88 A_663.8_ (2)

Chl_tot_ = (7.12 A_663.8_) + (17.67 A_646.8_) (3)

Car = (1000 A_480_ − 1.12 Chl a − 34.07 Chl b)/245 (4)

#### 4.3.2. Gas Exchange Parameters

Gas exchange parameters were measured non-destructively using an integrated fluorometer and gas exchange system (iFL; ADC Bioscientific Ltd., Hoddesdon, UK). Net photosynthetic rate (Pn), transpiration rate (E), and stomatal conductance (gs) were measured on fully expanded, photosynthetically mature leaves. Measurements were conducted between 8:00 and 13:00 UTC at an irradiance of 650 µmol m^−2^ s^−1^ and a leaf temperature of 25 °C, following Kuklová et al. [29].

#### 4.3.3. Chlorophyll Fluorescence Parameters

Chlorophyll fluorescence was measured on the same apple leaves used for gas exchange analysis. Parameters including minimum fluorescence (F_0_), maximum fluorescence (Fm), and variable fluorescence (Fv = Fm − F_0_) were recorded using the iFL system. Leaves were dark-adapted for 30 min prior to measurement. Fluorescence parameters Fv/Fm (maximum quantum efficiency of PSII) and Fv/F_0_ (potential photochemical efficiency) were calculated. Measurements were performed on five fully expanded upper leaves per tree using a saturating pulse of 3000 µmol m^−2^ s^−1^ and a measurement duration of 5 s.

#### 4.3.4. Leaf Water Status

Leaf water status (ψ_leaf;_ MPa) was determined using a dew point water potential meter (WP4C; Decagon Devices Inc., Pullman, WA, USA). Leaf samples were collected, sealed, and subsequently frozen (−18 °C) and thawed prior to measurement to obtain expressed sap. The obtained values should be interpreted as an approximation of leaf water status rather than absolute leaf water potential, as the freezing–thawing procedure may influence the measured values. However, this method enables consistent comparison of relative differences among treatments and genotypes.

### 4.4. Statistical Analysis

Four independent biological replicates per treatment were used. Data were analyzed using two-way ANOVA with genotype and irrigation treatment as main factors, followed by Tukey’s HSD post hoc test at *p* ≤ 0.05. Homogeneity of variance and normality were verified using Levene’s and Shapiro–Wilk tests. Linear regression analysis with dummy variables was applied to further assess genotype and treatments.

Measurements performed at different BBCH stages were evaluated independently for each sampling date to avoid pseudoreplication associated with repeated measurements on the same plants. This approach allowed comparison of treatment effects within each developmental stage. All statistical analyses were conducted using Statistical software (14.0; StatSoft, Tulsa, OK, USA). Results are presented as mean values ± standard deviation (SD). Standard deviation (SD) was used instead of the standard error of the mean (SEM) to better reflect biological variability among individual trees.

## 5. Conclusions

This study showed that short-term water deficit significantly influences the physiological performance of apple trees, with responses varying among genotypes. Changes were observed in gas exchange, pigment content, chlorophyll fluorescence, water use efficiency, and leaf water status, indicating that multiple physiological processes are affected by reduced water availability.

Genotype-dependent differences were evident, with some genotypes (e.g., ‘HL 2350’ and ‘B11’) maintaining relatively stable physiological parameters during water deficit conditions, while others (e.g., ‘Idared’ and ‘Rubinstep’) exhibited more pronounced responses. However, these findings are based on short-term measurements under semi-controlled container conditions and should be interpreted with caution.

Rehydration led to partial recovery of physiological parameters, suggesting that the effects of water deficit were not fully reversible within the experimental timeframe.

Overall, the results highlight the importance of considering genotype-specific physiological responses when studying drought stress in apple. The identified differences provide a basis for further investigation; however, future studies under field conditions, including growth, yield, and fruit quality traits, are necessary to determine the practical relevance of these findings.

## Data Availability

The original contributions presented in this study are included in the article. Further inquiries can be directed to the corresponding author.

## Supplementary Materials

The following supporting information can be downloaded at: https://www.mdpi.com/article/10.3390/plants15081179/s1, Figure S1. Gas exchange parameters in leaves of apple genotypes (‘Galaval’, ‘Idared’, ‘Rubinstep’, ‘B11’, ‘HL 308’, ‘HL 2010’, ‘HL 2350’, and ‘HL 827’) under control conditions, water deficit, and subsequent rehydration. (A) Water use efficiency (WUE; Pn/E), (B) stomatal conductance (gs; mol H_2_O m^−2^ s^−1^), and (C) transpiration rate (E; mmol H_2_O m^−2^ s^−1^). Bars represent mean ± standard deviation (SD) (*n* = 4). Figure S2. Relationships between selected physiological parameters and leaf water status in apple genotypes under different water regimes. Figure S3. Experimental setup in the foil-covered facility showing container-grown apple trees (‘Galaval’, ‘Idared’, ‘Rubinstep’, ‘B11’, ‘HL 308’, ‘HL 2010’, ‘HL 2350’, and ‘HL 827’) under semi-controlled conditions.
