# Supplementary figures and images for: Water Deficit and Rehydration Reveal Genotypic Differences in Apple Tree Physiological Performance
Source: Plants (Basel). 2026 Apr 10;15(8):1179. doi: 10.3390/plants15081179 (PMC13120257; doi:10.3390/plants15081179)

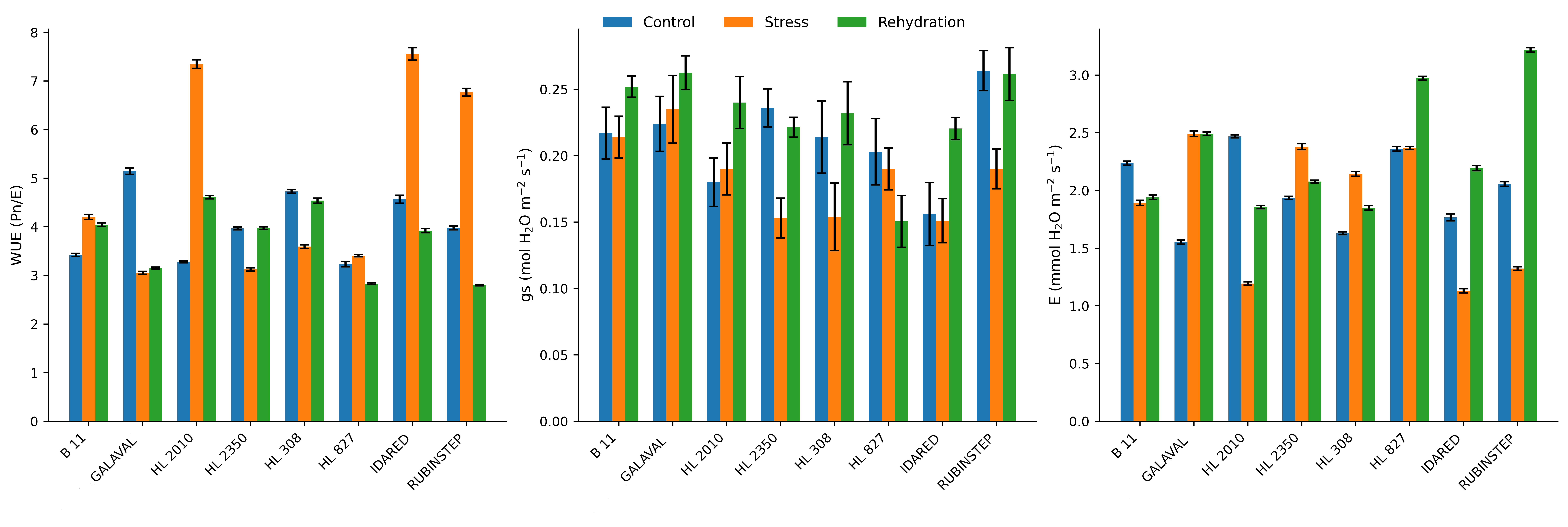

Supplement: Supplementary file 1 [file plants-15-01179-s001.zip › Supplementary_Figure_S1_GasExchange.tif]

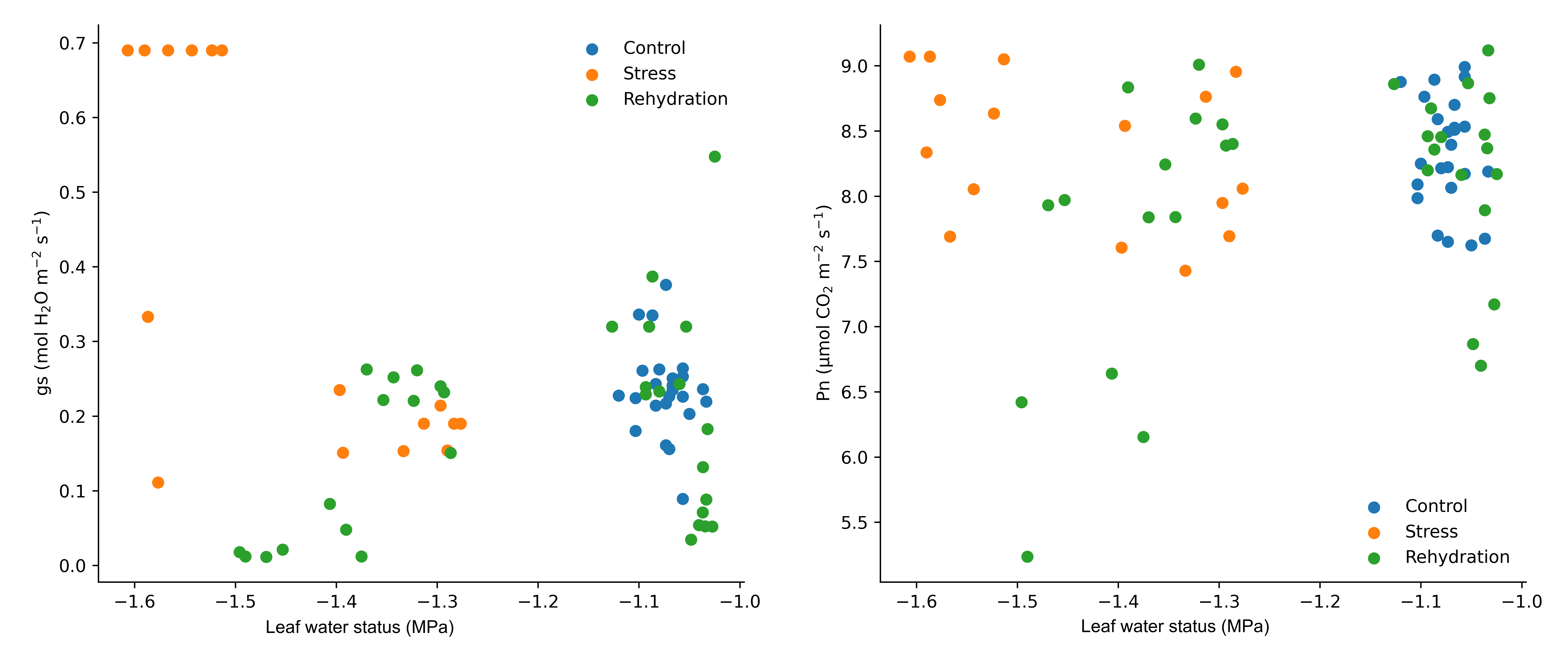

Supplement: Supplementary file 1 [file plants-15-01179-s001.zip › Supplementary_Figure_S2.tif]

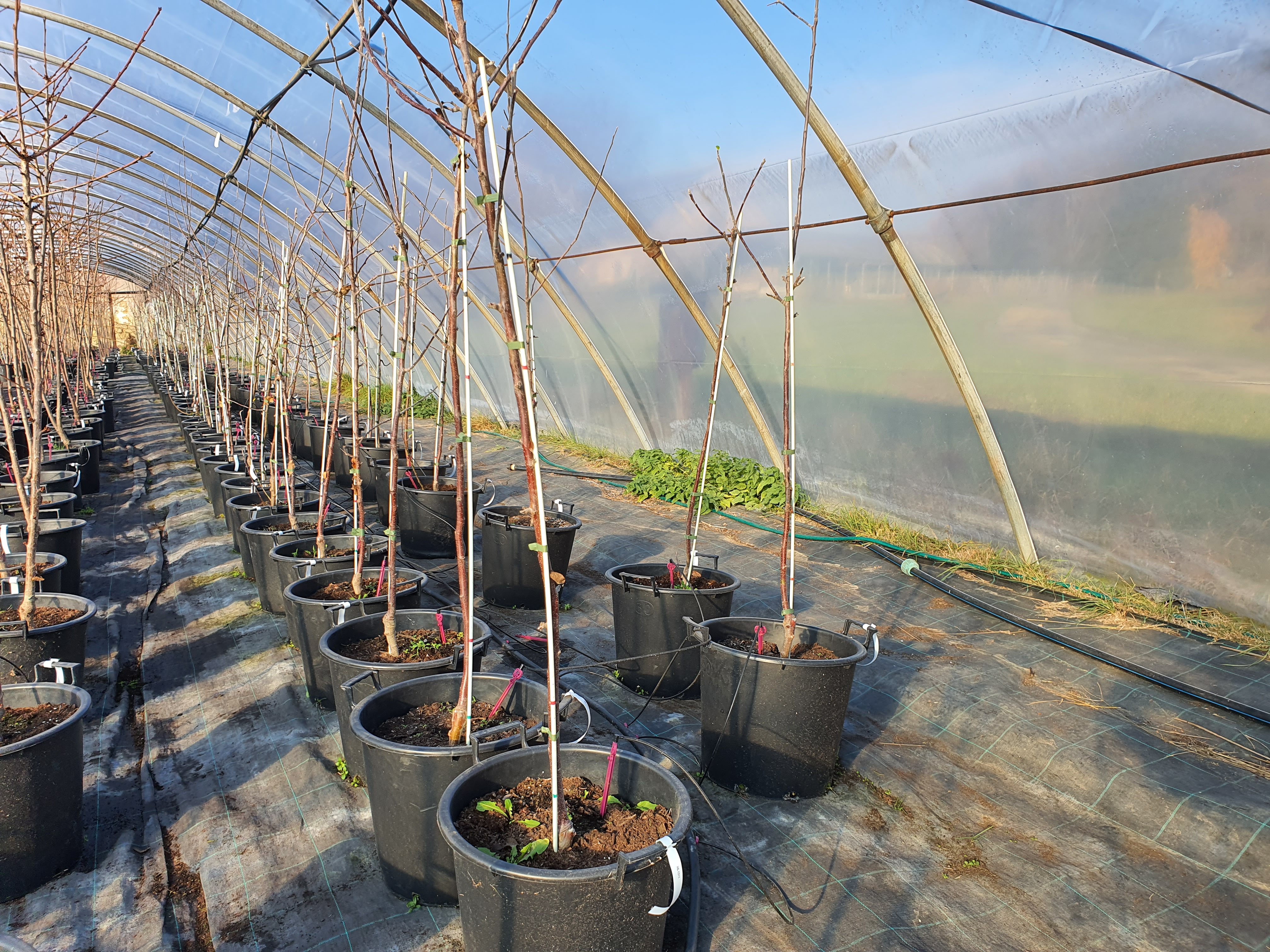

Supplement: Supplementary file 1 [file plants-15-01179-s001.zip › Supplementary_Figure_S3.jpg]
